# Effect of Hard-Segment Structure on the Properties of Polyurethane/Poly(Ethyl Methacrylate) Damping Composites

**DOI:** 10.3390/polym17050636

**Published:** 2025-02-27

**Authors:** Jinbao Ma, Chi Ma, Risheng Long, Yan Jiang, Xingjia Wang, Chang Liu, Fan Li, Lee Tin Sin

**Affiliations:** 1 School of Material Science and Engineering, Shenyang University of Chemical Technology, Shenyang Economic and Technological Development Zone, 11th Street, Shenyang 110142, China; r1732165542@163.com (J.M.); na_jiangyan@sina.com (Y.J.); w15040496303@163.com (X.W.); 13072499982@163.com (C.L.); 2 China-Spain Joint Laboratory on Material Science, Shenyang University of Chemical Technology, Shenyang Economic and Technological Development Zone, 11th Street, Shenyang 110142, China; 3 Equipment Reliability Institute, Shenyang University of Chemical Technology, Shenyang Economic and Technological Development Zone, 11th Street, Shenyang 110142, China; etomi@163.com; 4 School of Health Management, China Medical University, Shenyang North New Area, No. 77 Puhe Road, Shenyang 110122, China; fanli@cmu.edu.cn; 5 Department of Chemical Engineering, Lee Kong Chian Faculty of Engineering and Science, Universiti Tunku Abdul Rahman, Jalan Sungai Long, Bandar Sungai Long, Cheras, Kajang 43000, Malaysia; leets@utar.edu.my

**Keywords:** polyurethane, isocyanate, damping properties, microphase separation, molecular dynamics simulation

## Abstract

Damping material performance influences the efficacy of vibration and noise reduction. However, traditional damping materials often have low damping peaks or narrow damping temperature ranges. In this study, a series of polyurethane (PU)/poly(ethylene methacrylate) (PEMA) composites were synthesised, in which the PU hard segments were varied using toluene diisocyanate (TDI), diphenylmethane diisocyanate (MDI), isophorone diisocyanate (IPDI), and hexamethylene diisocyanate. The soft segments comprised tetrahydrofuran homopolymer glycol. The influence of the hard-segment structure on the properties of the PU/PEMA composites was investigated by infrared spectroscopy, thermogravimetric analysis, dynamic mechanical thermal analysis, and other experimental methods. The performance mechanism was explored from a molecular perspective via integration with molecular dynamics simulations. The PU/PEMA material with IPDI hard segments comprised numerous microphase-separated structures and exhibited greater free volume, fuller molecular-chain movement, and the highest damping performance, with a loss factor of 0.56. The PU/PEMA composites synthesised with TDI and MDI hard segments exhibited better compatibility, with the MDI-PU/PEMA system exhibiting a higher hydrogen-bonding force. This material also exhibited a higher thermal stability, with an initial breakdown temperature of 287.87 °C. This study provides a basis for regulating and optimising the performance of PU-based damping materials.

## 1. Introduction

If left unaddressed, vibration and noise problems can seriously affect the safe operation of equipment and the physical and mental health of people [1]. The application of damping materials is a primary strategy for mitigating vibration and noise, with their performance significantly influencing the efficacy of vibration and noise reduction [2,3,4]. As an important damping material, polyurethane (PU) has attracted wide attention owing to its unique soft-/hard-segment structure and molecular design flexibility [5,6,7]. Polyurethane damping materials have shown promise in a wide range of applications in the automotive, aerospace, and electronics sectors due to their excellent damping properties. Tang et al. [8]. designed a PU-based damping material comprising polycaprolactone diol and isophorone diisocyanate (IPDI). They introduced 4,4′-diamino diphenyl disulfide (DAPS) as a chain extender in PU and investigated its effect on the performance of PU elastomers. At 30% DAPS, the loss factor reached 0.43. Although PU materials have certain damping and wear-resistant properties, some limitations (such as low damping properties) exist when PU is used alone [9,10,11]. Therefore, methods like blending [12], copolymerising [13], and constructing interpenetrating polymer networks (IPNs) [14] are frequently employed to enhance the damping properties. PU-based damping materials with IPN structures have attracted the attention of some researchers owing to their forced inter-compatibility structure and straightforward preparation methods. Moreover, their structure is conducive to damping performance [15]. Yu et al. [16]. prepared an elastomer based on a PU/epoxy IPN, reporting that the IPN structure improved the thermal decomposition temperature and damping properties of the material. The peak loss factor reached 0.53, which was 129.6% higher than that of the PU material alone. Similarly, Moradi et al. [17]. prepared PU/poly(methyl methacrylate) (PMMA) foam with an IPN structure. The damping temperature domain increased and then decreased with increasing PMMA content, reaching its maximum at a PU/PMMA mass ratio of 3:1. However, most research on PU-based IPN damping materials focuses on the effect of the type and ratio of the two interpenetrating materials; few studies have explored the influence of the PU structure itself.

The soft and hard segments within PU significantly influence the formation of the IPN structure and its final performance. Although detailed microstructure–property analyses are important, performing such analyses is challenging using conventional experimental techniques and characterisation methods. Nevertheless, simulation technologies have flourished owing to advancements in computer technology, and molecular dynamics (MD) simulations have become an important methodology in materials science [18,19,20]. For example, Li et al. [21] investigated the effect of hydrogen bonding on the thermal and mechanical properties of epoxy resins by using MD simulations. They reported that the hydrogen bonding strength was inversely proportional to the free volume and had a limited effect on the glass transition temperature, which was more affected by molecular weight and steric hindrance.

In this study, PU/poly(ethyl methacrylate) (PEMA) IPN damping materials with various hard-segment structures were synthesised. By combining experimental research and MD simulations, the influence of the microphase structure on both damping performance and thermal stability was examined. Furthermore, the relationship between the microscopic interactions and damping performance at the molecular scale was investigated using MD simulations. These investigations establish a foundation for understanding the performance of PU-based damping materials and provide a reference for the regulation and optimisation of PU-based damping material performance.

## 2. Materials and Methods

### 2.1. Materials

Poly(tetrahydrofuran ether) glycol (PTMG; industrial grade, *M*_n_ = 1000) was supplied by Zhengzhou Alpha Chemical Co., Ltd. (Zhengzhou, China) Toluene diisocyanate (TDI; industrial grade), diphenylmethane diisocyanate (MDI, industrial grade), hexamethylene diisocyanate (HDI; industrial grade), and IPDI (industrial grade) were obtained from Shanghai Aladdin Biochemical Technology Co., Ltd. (Shanghai, China). Ethyl methacrylate (EMA; industrial grade) and azobisisobutyronitrile (AIBN; industrial grade) were provided by Shanghai McLean Biochemical Co., Ltd. (Shanghai, China), and 3,3′-dichloro-4,4′-diaminodiphenylmethane (MOCA; industrial grade) was provided by Suzhou Xiangyuan Chemical Co., Ltd. (Suzhou, China).

### 2.2. Synthesis Procedures

**Figure 1:**
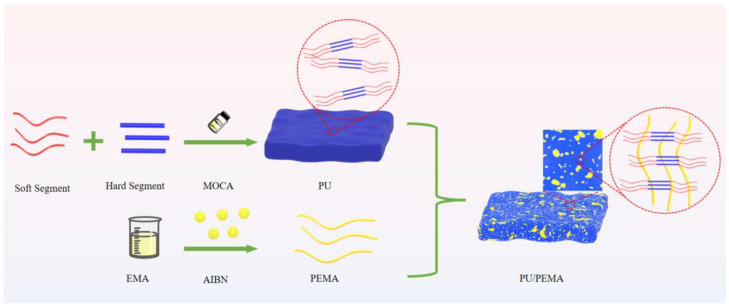
Schematic of polyurethane (PU)/poly(ethyl methacrylate) (PEMA) damping composite synthesis.

PU/PEMA damping composites with different hard-segment structures were synthesised as follows (Figure 1). First, PTMG was vacuum dehydrated for 3 h at 110 °C. Various isocyanates were added, and the mixtures were reacted for 3–4 h at 70–80 °C. A titration method was employed to determine the isocyanate content, which was the theoretical value of reaction termination. The PU pre-polymer was added to EMA at a PU/EMA mass ratio of 6:4 and evenly mixed. Next, add 0.4% by weight of EMA as initiator AIBN and stir for 5 min. MOCA was then added with stirring for 5 min. The resulting mixture was vacuum defoamed for 5 min and then poured into preheated polytetrafluoroethylene moulds. Finally, curing was conducted at 80 °C for 24 h, followed by resting at 25 °C for one week to produce PU/PEMA IPN damping materials with different hard-segment structures.

### 2.3. Characterisation

#### 2.3.1. Fourier-Transform Infrared Spectroscopy

A Nexus 470 infrared spectrometer (TA, Delaware, USA) was used for Fourier-transform infrared (FTIR) spectroscopy in the attenuated total reflection mode. Spectra were collected within the wavenumber range of 4000–500 cm^−1^ with a scan number of 16. The samples for infrared examination were thin enough to fall within the absorbance region where the Beer–Lambert law was followed [22]. The curve-fitting procedure was carried out using the software “OMNIC 8.2”. All infrared spectra acquired through the experiment were normalised.

#### 2.3.2. Thermogravimetric Analysis

Thermogravimetric analysis (TGA) was conducted in a nitrogen atmosphere using an STA 449 F3 thermal analyser (Netzsch, Bavaria, Germany). The temperature range and heating rate were 25–600 °C and 10 °C/min, respectively.

#### 2.3.3. Dynamic Mechanical Analysis

Dynamic mechanical analysis (DMA) was performed using a diamond-type dynamic viscoelastic spectrometer (PerkinElmer, Waltham, MA, USA). The testing temperature, heating rate, and vibration frequency were −80 to 160 °C, 3 °C/min, and 1 Hz, respectively.

#### 2.3.4. Scanning Electron Microscopy

Scanning electron microscopy (SEM; JSM-IT800, Nippon Electron Corporation, Tokyo, Japan) was used for sample analysis. The samples were freeze-fractured and gold-coated prior to observation.

#### 2.3.5. Atomic Force Microscopy

Atomic force microscopy (AFM; Bruker, Ettlingen, Germany) was used for sample analysis at a spring constant of 40 N m^−1^ and resonance frequency of 300 kHz.

### 2.4. MD Simulation

**Figure 2:**
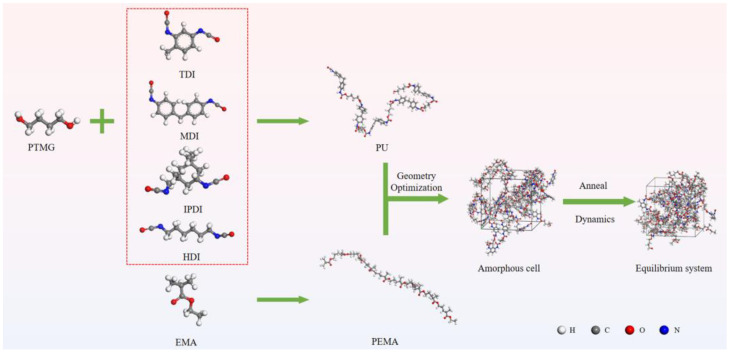
Flow chart of molecular dynamics (MD) simulations.

MD simulations were performed using Materials Studio (Figure 2). First, PU and PEMA repeating units were constructed to model the molecular chains, and structural optimisation was performed in 100,000 steps. Subsequently, the Amorphous Cell module was used to construct the amorphous cell structures of PU and PEMA with different hard-segment structures at a PU/PEMA mass ratio of 6:4, and 100,000 steps of structural optimisation were performed. The lowest-energy structures were annealed in the temperature range of 300–600 K. Equilibrium treatment was conducted using the canonical ensemble (NVT) for 500 ps, and kinetic calculations were performed using the isothermal and isobaric (NPT) and NVT systems for 200 ps. Hydrogen bonding, the free volume fraction, and the radial distribution function of the equilibrium system were analysed after relaxation.

#### 2.4.1. Hydrogen Bonding Analysis

Hydrogen bond formation affects the properties of PU. Two types of hydrogen bond exist within the PU/PEMA composite system: intramolecular hydrogen bonds between two urethane esters and intermolecular hydrogen bonds between urethane and acrylate esters. The latter ten-frame model of the equilibrium system was computationally analysed using a hydrogen bonding script.

#### 2.4.2. Free Volume Fraction

Free volume refers to the voids created when molecules stack in a certain arrangement [23]. These voids provide space for molecular movement, resulting in excellent mechanical properties of the material. The free volume fraction (FFV) is used to indicate the percentage of free volume and is calculated as follows:

(1) $$FFV=\frac{V_{f}}{V_{f}+V_{o}}$$

where *V*_f_ and *V*_o_ are the free and occupied volumes, respectively.

#### 2.4.3. Radial Distribution Function

The radial distribution function (RDF) is used to characterise the aggregation pattern of a molecule and indicates the probability of an atom appearing to other atoms within a radius *r* [24]. The truncation radius, which should be less than one-half of the minimum side length of the system, is selected, and the RDF (*g*(*r*)) is calculated as follows:

(2) $$g\left(r\right)=\frac{N\left(r,\;\;(r+dr)\right)}{4\pi r^{2}dr\rho }$$

where *N*(*r*,(*r* + d*r*)) is the number of particles with a distance from the reference point *r* to a point *r* + d*r*, and *ρ* is the density of the system.

## 3. Results and Discussion

### 3.1. Experimental Analyses

#### 3.1.1. Bonding Structure

**Figure 3:**
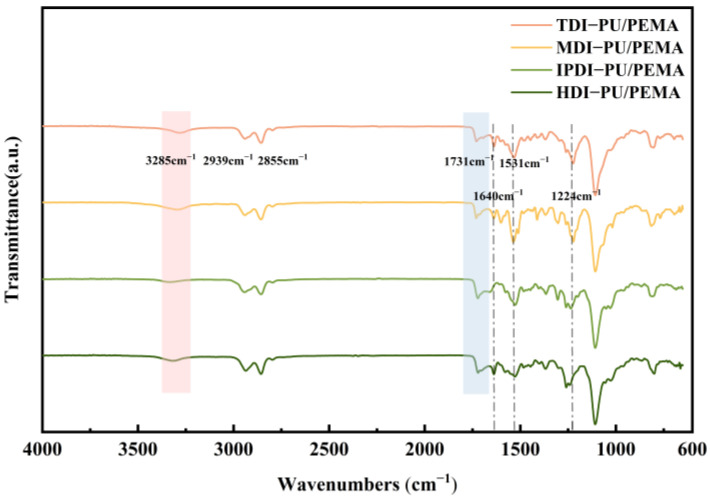
Fourier-transform infrared (FTIR) spectra of PU/PEMA composites with different hard segments.

Figure 3 shows the FTIR spectra of the PU/PEMA composites with different hard-segment structures. The absorption peaks at 3285 cm^−1^ were attributed to the contraction vibration peaks of N–H, whereas those at 2939 and 2855 cm^−1^ were attributed to the contraction vibrations of methyl and methylene groups, respectively. These absorption peaks were stronger for the composite with HDI hard segments because HDI comprises six methylene groups. The peaks at 1531 and 1224 cm^−1^ correspond to the characteristic absorption peaks of urethane esters and C–O bonds, respectively, in MOCA. The quadruple peaks at 1350–1470 cm^−1^ in the TDI- and MDI-PU/PEMA systems correspond to the bending vibrations of methyl and methylene groups in the benzene ring, respectively. No characteristic peaks appeared near 2270 cm^−1^, indicating that the different isocyanates completely reacted; therefore, PU preparation was successful in all cases. The carbonyl (C=O) vibration peaks of urethane and acrylate appeared at 1731 cm^−1^. Notably, the addition of PEMA increased the number of C=O groups in the system, and the C=C absorption peak of EMA appeared at 1640 cm^−1^, confirming that the PU/PEMA systems were successfully synthesised.

**Figure 4:**
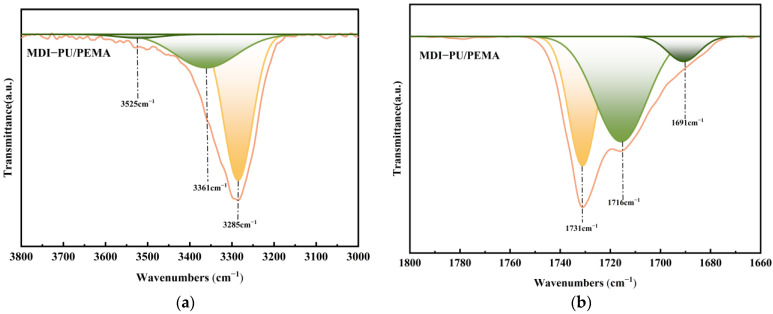
Characteristic peak areas of diphenylmethane diisocyanate (MDI)-based PU/PEMA: (**a**) N–H; (**b**) C=O.

To comprehensively investigate the influence of intermolecular forces on the properties of the PU/PEMA composite systems, changes in hydrogen bonding were examined by fitting the differences in specific peak areas. Figure 4 shows this process using MDI-PU/PEMA as an example. Intramolecular hydrogen bonds among urethanes and intermolecular hydrogen bonds between urethanes and acrylates were the two most dominant types of hydrogen bonding in the PU/PEMA composite system. Figure 4a,b shows free N–H and C=O at 3285 and 1731 cm^−1^, urethane intramolecular hydrogen bonding at 3361 and 1716 cm^−1^, and urethane–acrylate intermolecular hydrogen bonding at 3525 and 1691 cm^−1^. Hydrogen bonding formation broadened the infrared absorption peaks of N–H and C=O and yielded two small shoulder peaks. The peak area was slightly larger for intermolecular hydrogen bonds due to the difference in the strength between the two hydrogen bonding types [25,26].

The percentage of each type of hydrogen bond was calculated as the ratio of the N–H or C=O peak area to the total peak area of hydrogen bonding interactions, expressed as *X*_(N–H)_ and *X*_(C=O)_, respectively [27], using the following formula:

(3) $$X_{\left(N–H\right)}\;or\;X_{\left(C=O\right)}=\frac{S_{b}}{S_{b}+S_{f}}$$

where *S*_b_ and *S*_f_ are hydrogen-bonded N–H (or C=O) and free N–H (or C=O), respectively.

The peak patterns of the four systems were fitted separately with split peaks, and the calculated results are listed in Table 1. The MDI-PU/PEMA system exhibited the strongest peaks for both types of hydrogen bond, indicating that it had the most compact internal network.

#### 3.1.2. Thermal Stability

**Figure 5:**
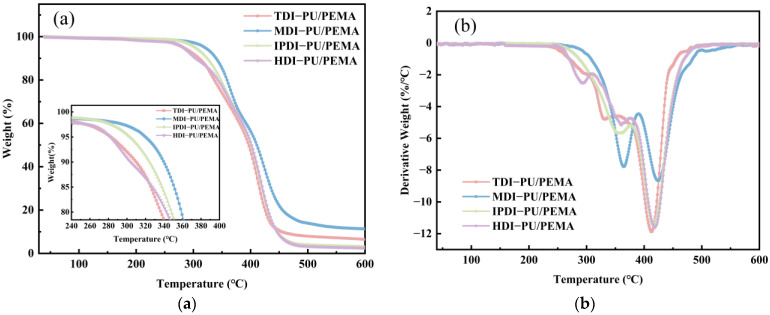
Thermogravimetric analysis (TGA) results of PU/PEMA composites with different hard segments: (**a**) TGA curves; (**b**) differential thermogravimetry (DTG) curves.

Figure 5 shows the thermogravimetry results of the PU/PEMA composites with different hard segments, and Table 2 lists the relevant data. At temperatures below 200 °C, no thermal weight loss was observed in the four systems, as shown in the TGA curves in Figure 5a. However, different decomposition processes were observed above 200 °C. Among the four systems, MDI-PU/PEMA exhibited the highest initial decomposition temperature and thermal stability owing to the two benzene rings in the MDI molecular chain. These rings provided additional conjugate stability, thereby resisting thermal decomposition to a certain extent. Because the MDI molecular structure is more symmetrical and regular, more hydrogen bonding interactions occurred within this system. This resulted in a more stable mesh structure with PEMA and better thermal resistance. When sample decomposition was complete, the MDI-PU/PEMA system exhibited the highest residual carbon rate of 11.35%. The higher stability of the benzene ring means that it is more likely to form a carbon layer during thermal decomposition, thereby increasing the residual carbon rate. IPDI has a cyclic structure in its molecular chain that is more stable than the straight-chain structure of HDI; therefore, the initial decomposition temperature of IPDI-PU/PEMA was higher than that of HDI-PU/PEMA. Because both IPDI and HDI are aliphatic isocyanates and do not have rigid structures, their residual carbon rates were low (3.14% and 2.69%, respectively).

The differential thermogravimetry (DTG) curves shown in Figure 5b revealed two thermal decomposition stages in the TDI-, MDI-, and IPDI-PU/PEMA systems and three in the HDI-PU/PEMA system. The first stage occurred at 288–390 or 252–377 °C, which corresponds to the decomposition of the isocyanates in PU via C–N bond breakage. Owing to the relatively high intermolecular forces in these composites, isocyanate and PEMA decomposed simultaneously in this temperature range. The second stage occurred between 390–500 and 377–500 °C, indicating the rapid decomposition of polyol in PU. The third decomposition stage, which only existed in HDI-PU/PEMA, occurred at 247–305 °C and corresponded to partial decomposition. Although IPN systems can overcome incompatibility issues, the compatibility between HDI-PU and PEMA is poor. Because certain independent phases had poor thermal stability, early decomposition occurred.

#### 3.1.3. Damping Performance

**Figure 6:**
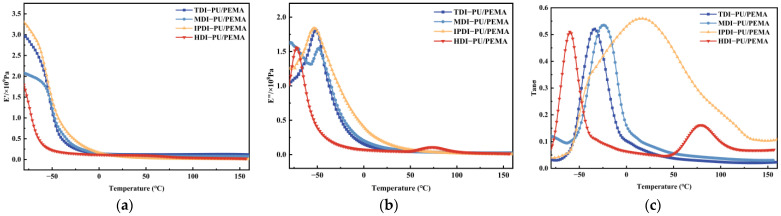
Dynamic mechanical analysis (DMA) curves of PU/PEMA with different hard segments: (**a**) *E*′; (**b**) *E*″; (**c**) tan*σ*.

Figure 6a presents the energy storage modulus (*E*′) versus temperature curves for the PU/PEMA composites with different hard segments [28]. The energy storage modulus of each sample decreased with increasing temperature. The IPDI-PU/PEMA system exhibited the highest energy storage modulus of 3.2 GPa.

Figure 6b shows the material loss modulus (*E*″) versus temperature curves. The loss modulus peaks in the glass transition region. For the IPDI-PU/PEMA system, the loss modulus peaked at −53 °C; therefore, the material dissipated the most energy at this temperature and converted more mechanical energy into thermal energy during the dynamic process. The molecular chain of MDI-PU was the most rigid, hindering the movement of the chain segments; therefore, MDI-PU/PEMA had the highest glass transition temperature. The molecular chain of HDI-PU was more flexible; therefore, HDI-PU/PEMA exhibited the lowest glass transition temperature. Due to the relatively poor compatibility between HDI-PU and PEMA, a double peak was observed at 75 °C.

The loss factor (tan*σ*) is a measure of the energy lost by a material when resisting external vibration; therefore, it reflects the damping performance of the material. Figure 6c shows that the IPDI system-PU/PEMA exhibited the highest loss factor, and the damping curve exhibited a single-peak profile. Several shoulder peaks were observed at −37 and 79 °C, which effectively expanded the damping temperature range of the material. Thus, more microphase separation regions may have formed inside the material. Moreover, the broad peak phenomenon observed in the DMA curves indicates that different microregions underwent molecular-chain disentanglement at different temperatures. The damping curves of the MDI- and TDI-PU/PEMA systems exhibited only one peak, indicating the good compatibility of the materials. This result was likely due to π–π stacking between the benzene ring and PEMA and other non-covalent interactions that enhanced compatibility. The HDI-PU/PEMA damping curve revealed two completely independent peaks, indicating pronounced microphase separation. Thus, the compatibility between HDI-PU and PEMA was relatively poor, and the internal hydrogen bonding force of the material was weaker.

#### 3.1.4. Microstructure Analysis

**Figure 7:**
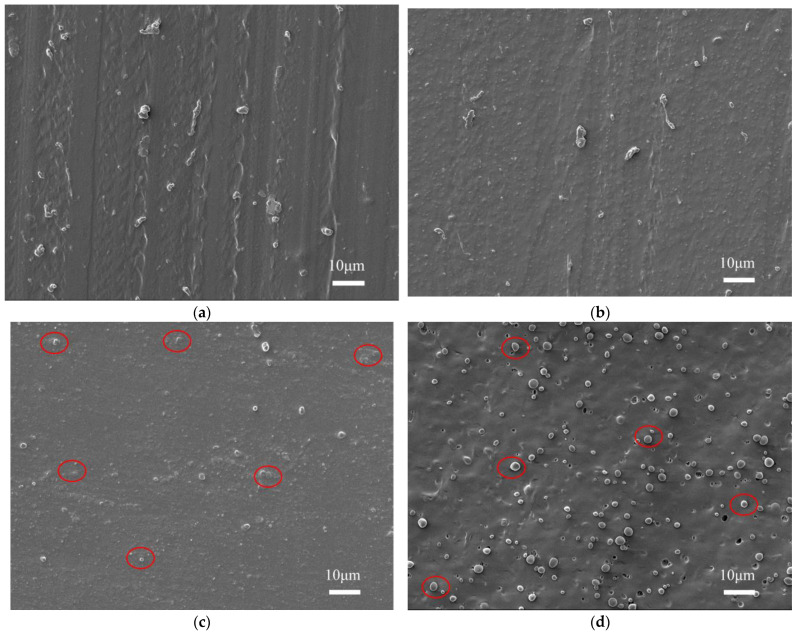
Scanning electron microscopy (SEM) images of PU/PEMA composites with different hard segments: (**a**) TDI-based PU/PEMA; (**b**) MDI-PU/PEMA; (**c**) IPDI-based PU/PEMA; (**d**) HDI-based PU/PEMA.

Owing to the unique structure of PU, the contact area between the dispersed and continuous phases is relatively small; therefore, microphase separation can occur. The degree of microphase separation in PU affects its performance and application. The micromorphology of the sample segments was observed using SEM (Figure 7). PU constituted the continuous phase, whereas PEMA formed a dispersed phase. As shown in Figure 7a,b, PEMA and PU did not exhibit microphase separation in the TDI- and MDI-PU/PEMA systems. Compared to the TDI-PU/PEMA system, the MDI-PU/PEMA system had a more regular molecular-chain structure, resulting in a homogeneous cross-sectional surface. The IPDI-PU/PEMA system exhibited partial microphase separation and additional microphase structures. The presence of more protrusions on the surface of the IPDI system represents the presence of another phase, and this uneven distribution of different phases on the surface of the material is a visual representation of microphase separation. This phenomenon is more evident in Figure 7d. The presence of another phase is shown in the red areas of Figure 7c,d. The HDI-PU molecular chain is more flexible, and its glass transition temperature is lower than that of PEMA. Therefore, the compatibility between HDI-PU and PEMA was not good, resulting in microphase separation, which corresponded to the bimodal phenomenon in the DMA results.

**Figure 8:**
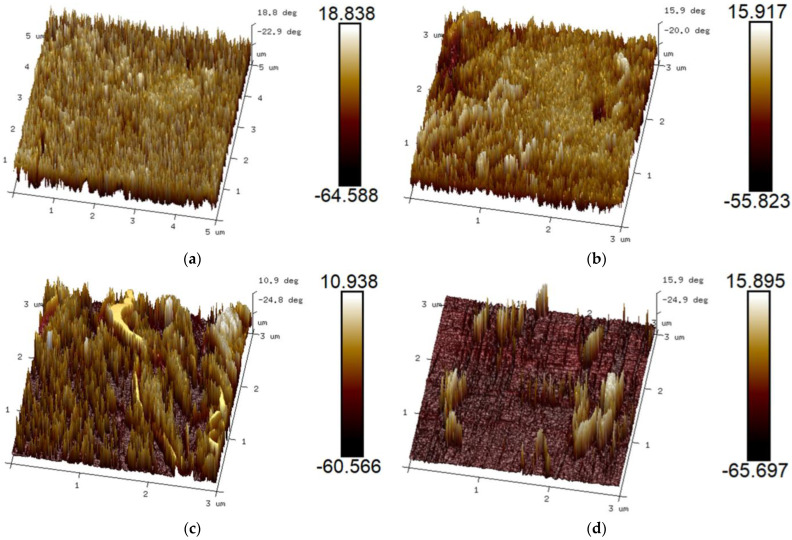
Atomic force microscopy (AFM) phase diagrams of PU/PEMA composites with different hard segments: (**a**) TDI-PU/PEMA; (**b**) MDI-PU/PEMA; (**c**) IPDI-PU/PEMA; (**d**) HDI-PU/PEMA.

Figure 8 shows the AFM phase diagrams of the PU/PEMA composites with different hard segments. The hard segments exhibited a higher modulus (brighter colours), whereas the soft segments exhibited a lower modulus (darker colours). As shown in Figure 8, the TDI- and MDI-PU/PEMA systems exhibited relatively weak microphase separation, whereas the IPDI- and HDI-PU/PEMA systems showed greater microphase separation. TDI and MDI are aromatic isocyanates with rigid benzene rings in the molecular chain that increase their rigidity. The presence of benzene rings hindered microphase separation to a certain extent. Because IPDI and HDI are aliphatic isocyanates, the flexibility of the molecular chain was greater. Thus, the soft and hard segments were more likely to become entangled, forming a microphase structure. Moreover, the phase domain structure was relatively small, and the degree of microphase separation was greater. In the IPDI-PU/PEMA system, the soft- and hard-segment phases were evenly distributed and formed microphase regions. In contrast, HDI has a linear structure, and the synthesised PU was more flexible and less compatible with PEMA, resulting in stronger microphase separation.

### 3.2. MD Simulation Results

#### 3.2.1. Hydrogen Bonding

**Figure 9:**
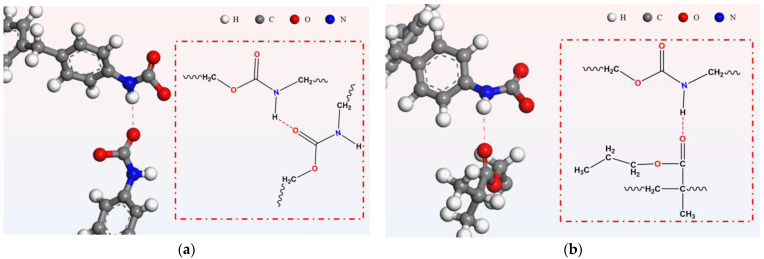
Schematic of hydrogen bonding in the MDI-PU/PEMA system: (**a**) intramolecular hydrogen bonding; (**b**) intermolecular hydrogen bonding.

**Figure 10:**
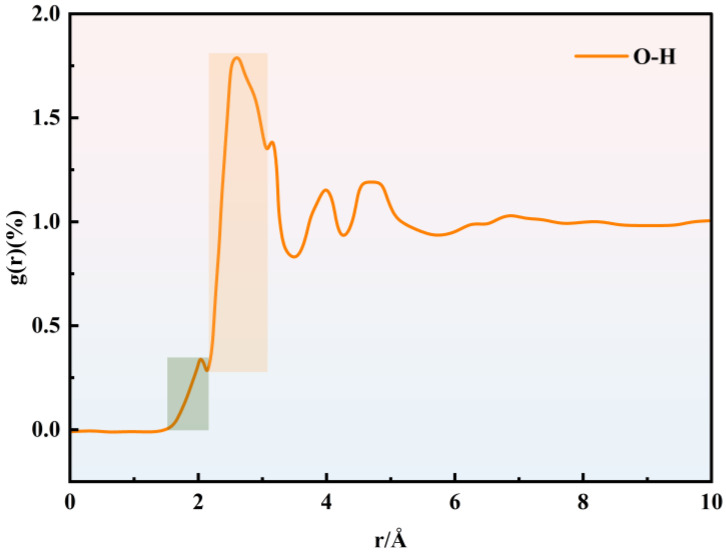
Radial distribution function (RDF) curves of O–H bonding in the MDI-PU/PEMA system.

The two primary types of hydrogen bonds in the PU/PEMA composite system, using the MDI-PU/PEMA system as an example, are shown in Figure 9. Figure 9a shows the intramolecular hydrogen bonding interactions within PU. These bonds were primarily formed between the oxygen atom (acceptor) of the carbonyl group in urethane and the hydrogen atom (donor) of the amino group. Figure 9b shows the intermolecular hydrogen bonding interactions between PU and PEMA. These bonds were primarily formed between the oxygen atom (acceptor) of the carbonyl group in PEMA and the hydrogen atom (donor) of the amino group in PU. The difference in the forces was primarily caused by steric hindrance. Intramolecular hydrogen bonds were constrained by the molecular structure when they formed, with a smaller spacing between atoms and shorter hydrogen bonds. In contrast, when intermolecular hydrogen bonds formed between two molecules, the steric hindrance was smaller, resulting in less restriction between atoms; thus, the hydrogen bonds were longer. Figure 10 shows the RDF curve between the O and H atoms involved in the formation of hydrogen bonds in the MDI-PU/PEMA system, from which the bond lengths of the two hydrogen bonds were determined [29]. In Figure 10, the green area represents intramolecular hydrogen bonding of polyurethanes and the orange area represents intermolecular hydrogen bonding between polyurethanes and acrylates. The peak pattern indicated that the effect of intermolecular hydrogen bonding was much larger than that of intramolecular hydrogen bonding. Thus, the number of intermolecular hydrogen bonds was greater, and the bonding energy was higher because the intermolecular hydrogen bonds had more space to adjust the spacing and angle of the atoms. Moreover, the formation of hydrogen bonds was closer to the ideal state, resulting in stronger interactions.

Table 3 presents the number of hydrogen bonds of each type. The MDI-PU/PEMA system had the highest number of intramolecular hydrogen bonding interactions, which was attributed to the double benzene ring structure of MDI that limited the molecular-chain conformation to a certain extent. Because the distance between the urethane groups was relatively fixed, the possibility of the carbonyl and amino groups being close to each other increased, rendering it more likely to induce intramolecular hydrogen bonding. Although intramolecular hydrogen bonding primarily affected the stability of the molecule, and the number and forces were small, the most crucial factor affecting the performance of the PU/PEMA system was intermolecular hydrogen bonding. As shown in Table 3, the number of intermolecular hydrogen bonds in the four systems followed the trend MDI > IPDI > TDI > HDI. The MDI-PU/PEMA system had better regularity, and the rigidity of the benzene ring stabilised the molecular conformation, rendering it easier to generate hydrogen bonds between PU and PEMA. TDI has a specific ring structure that is relatively complex, and the molecular chain of TDI-PU is not as regular as that of MDI-PU; therefore, the efficiency of hydrogen bonding was relatively low. The methyl group in the molecular chain of TDI increases the spatial resistance, which affects the formation of hydrogen bonds when it interpenetrates PEMA. The molecular chain of HDI-PU exhibited greater flexibility, rendering the spatial distribution of the molecular chain more chaotic and inconducive to the formation of hydrogen bonds. According to the macro-performance analysis, the MDI-PU/PEMA system had more hydrogen bonds. Moreover, the higher the bonding degree of the composite system, the more difficult it is to decompose; therefore, this system also had a higher thermal decomposition temperature. However, the hydrogen bond strength and effect of the TDI- and HDI-PU/PEMA systems were weaker; therefore, the molecular chains were easier to break, and the thermal decomposition temperature was lower. Moreover, the number and strength of hydrogen bonds affected the compatibility of the polymers to a certain extent. In general, more hydrogen bonding results in stronger attraction between two phases, enhancing the compatibility. It can also maintain connections between phases that tend to separate; thus, hydrogen bonding inhibits microphase separation.

#### 3.2.2. Free Volume Fraction Analysis

**Figure 11:**
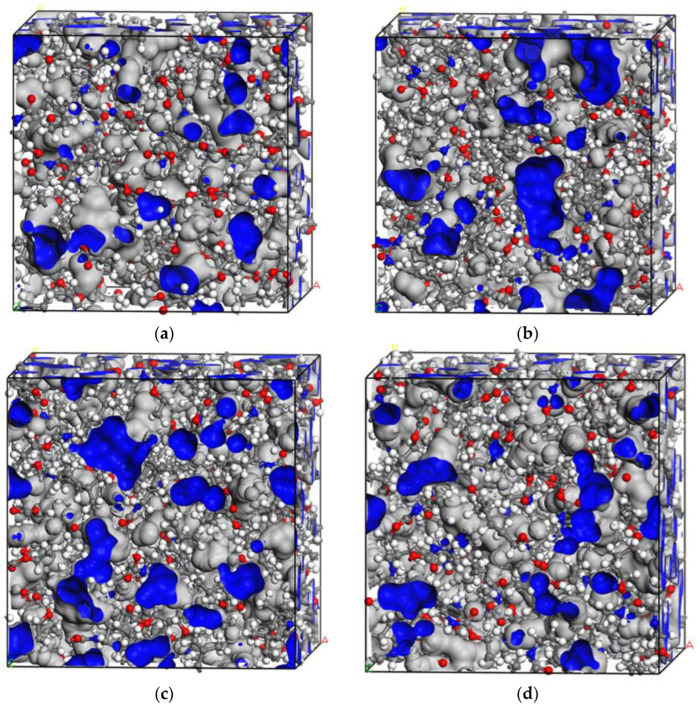
Free volume of PU/PEMA composites with different hard segments: (**a**) TDI-PU/PEMA; (**b**) MDI-PU/PEMA; (**c**) IPDI-PU/PEMA; and (**d**) HDI-PU/PEMA.

**Figure 12:**
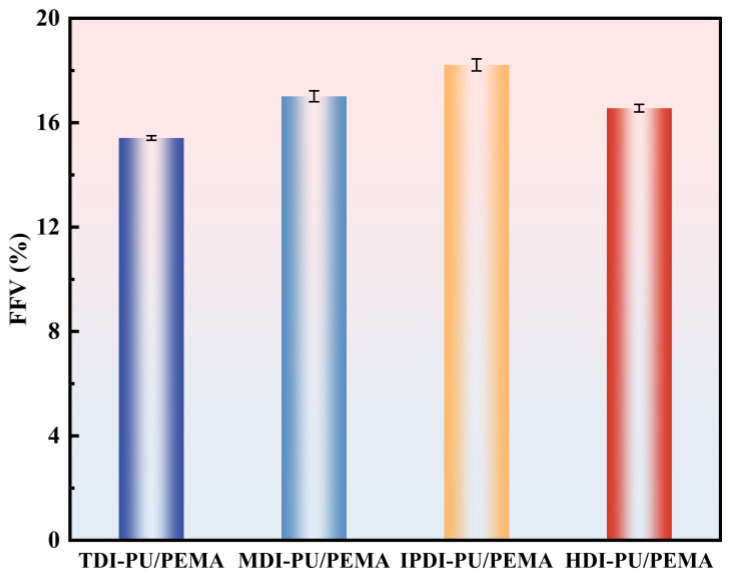
Free volume fraction statistics of the PU/PEMA composites with different hard segments.

The free volumes and free volume fractions of the PU/PEMA composite systems with different hard segments are shown in Figure 11 and Figure 12. The free volume fractions of the last three models of the equilibrium system were averaged and analysed to ensure the accuracy of the calculated results. Figure 12 shows that the free volume size followed the trend IPDI > MDI > HDI > TDI. The ring structure of IPDI caused its PU chain segment to exhibit higher steric hindrance. When forming an IPN with PEMA, more gaps were produced, and the range of molecular-chain movement increased; thus, the free volume fraction was higher, and molecules had more space for movement. MDI-PU molecular chains are more regular; therefore, they form neater arrangements with closer stacking in the IPN. Consequently, the hydrogen bonding force in the MDI-PU/PEMA system was higher, thereby restricting the molecular-chain movement and decreasing the free volume to below that of the IPDI-PU/PEMA system. However, the two benzene rings in MDI played a supportive role in the interpenetrating molecular chain; thus, the free volume was larger than that of the HDI- and TDI-PU/PEMA systems. The longer methylene chain of HDI resulted in the entanglement of molecular chains, and the spacing of the molecular chains decreased, thereby decreasing the free volume fraction. TDI has only one benzene ring structure, and its synthesised PU is less regular, which limits the movement of its molecular chains. Thus, the molecular chains did not flexibly adjust when forming interpenetrating structures, and the chains aligned closely with each other. Moreover, the interface between separated microphases in the TDI-PU/PEMA system was more regular, and the molecular chains were closely stacked at the phase interface, resulting in a low free volume. From the perspective of mechanical properties, a low free volume corresponds to less space between molecular chains. When the material is subjected to external forces, molecular chains can absorb energy through movement or rotation. Consequently, a higher free volume enables the molecular-chain segments to have more space to move, which is more effective in converting mechanical energy into thermal energy and results in better damping performance.

#### 3.2.3. Radial Distribution Function Analysis

**Figure 13:**
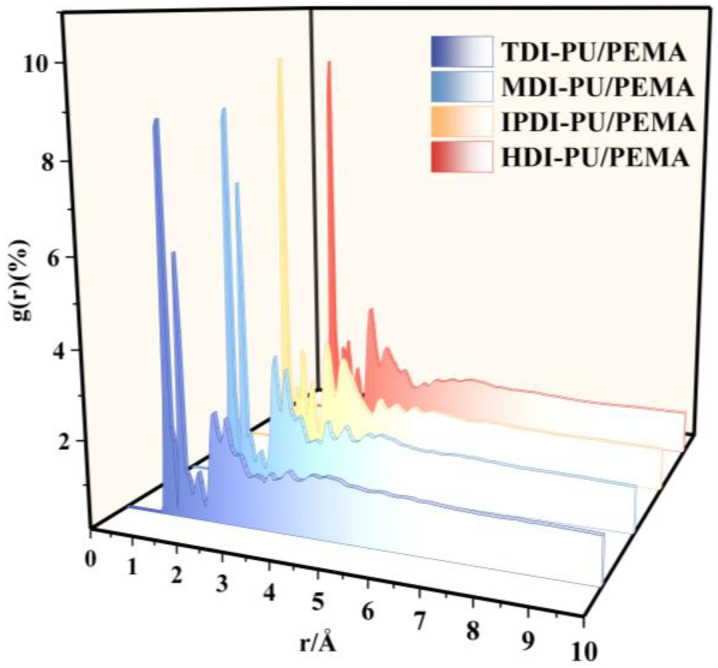
RDF curves of PU/PEMA with different hard segments.

Figure 13 shows the RDF curves of the PU/PEMA composites with different hard-segment structures. The first peak occurred at a truncation radius of 1.125 Å in all four systems, implying that the density of particles within the radius of 1.125 Å was the highest. Moreover, the particles were close together and closely stacked, with strong interactions. Within this truncation radius, covalent bonding played the primary role, and chemical bonding through shared electron pairs was stronger than other forces. The peaks and valleys at 1.4–1.5 Å indicated that the interactions between the particles were weak, corresponding to the low-density region of the substance. The peaks at 1.5–3.1 and 3.1–6.0 Å correspond to hydrogen bonding and van der Waals forces, respectively. These forces function at a greater distance from the central atom and are weaker than other chemical bonds. Moreover, no peak was observed above 6 Å in all four systems, indicating that the molecular chains were short-range disordered and amorphous. As the radius increased, the particle distribution became uniform and equal to the average density of the entire system, and the RDF value was approximately one.

The first peak appeared at a slightly higher RDF value for aliphatic isocyanates than for aromatic isocyanates. In particular, the first peak of the IPDI-PU/PEMA system was high and sharp, with the highest density and strong covalent bonding. This was because the spatial potential resistance of the IPDI structure was the largest, which improved the covalent bonding stability and force. The IPDI-PU/PEMA structure exhibits the largest steric hindrance, which enhances the covalent bonding stability and increases the covalent bonding strength. Covalent bonds are less likely to break and can be rotated and twisted to a certain extent when stretched or deformed by external forces. This enables the material to absorb more energy without breaking; thus, the tensile strength and toughness of the IPDI-PU/PEMA system were higher.

The second peak at 1.375 Å in the TDI- and MDI-PU/PEMA systems was due to the presence of the benzene ring, which resulted in a greater degree of aggregation, and the complex interactions such as π–π stacking introduced by the benzene ring. These interactions interfered with the distribution pattern of the atoms and resulted in a looser distribution, thereby reducing the value of the first peak.

## 4. Conclusions

In this study, different isocyanates were used to synthesise PU materials with different hard segments, which were combined with PEMA to form IPN structures. The thermal, damping, and microstructural properties of the polymers were analysed using TGA, DMA, SEM, and AFM. The effects of the structural differences of the PU hard segments on the damping properties of the PU/PEMA composites were systematically analysed from a molecular standpoint in conjunction with MD simulations. The results revealed that the aromatic isocyanate-synthesised PU had better compatibility with PEMA. In particular, the MDI-PU/PEMA system exhibited stronger hydrogen bonding, formed a more stable cross-linked network structure, and exhibited the highest thermal stability. The IPDI-PU/PEMA system had more phase regions inside the material, which broadened the damping temperature domain. The MD simulation results revealed that the IPDI-PU/PEMA system had more free volume and more adequate molecular-chain movement, resulting in better damping performance. The HDI-PU/PEMA system had poor material compatibility owing to the large difference in glass transition temperature between HDI-PU and PEMA, and the AFM results indicated that it had the largest degree of microphase separation. Investigating the effects of isocyanate type on the properties of PU/PEMA damping composites provides a reference for designing and preparing high-performance polymer damping materials.

## Figures and Tables

**Table 1:** Characteristic peak areas and *X*_(N–H)_ and *X*_(C=O)_ values for PU/PEMA composites with different hard segments.

Name of Samples	Area of Free N–H Peak (3285 cm^−1^)	Area of H-Bonded N–H Peak (3361 cm^−1^ and 3525 cm^−1^)	Area of Free C=O Peak (1731 cm^−1^)	Area of H-Bonded C=O Peak (1716 cm^−1^ and 1691 cm^−1^)	X(N–H) (%)	Error (%)	X(C=O) (%)	Error (%)
TDI-PU/PEMA	619.83	208.01	218.34	298.06	25.13	±3.28	57.72	±3.34
MDI-PU/PEMA	660.74	260.84	180.48	313.69	28.30	±3.19	63.48	±2.81
IPDI-PU/PEMA	280.90	110.77	505.83	778.21	28.28	±3.87	60.61	±3.09
HDI-PU/PEMA	511.50	143.44	248.39	332.34	21.90	±4.13	57.23	±3.96

**Table 2:** TGA data for PU/PEMA with different hard segments.

Name of Samples	T_onset_/°C	Carbon Yield (600 °C)%
TDI-PU/PEMA	236.11	6.52
MDI-PU/PEMA	287.87	11.35
IPDI-PU/PEMA	252.31	3.14
HDI-PU/PEMA	247.17	2.69

**Table 3:** Hydrogen bonding statistics of PU/PEMA composite systems with different hard segments.

Name of Samples	1.5–2.2/Å	2.2–3.1/Å
TDI-PU/PEMA	3	22
MDI-PU/PEMA	16	25
IPDI-PU/PEMA	6	24
HDI-PU/PEMA	3	19

## Data Availability

All relevant data are within the manuscrip.

## Abbreviations

The following abbreviations are used in this manuscript:

AFM	Atomic force microscopy
AIBN	Azobisisobutyronitrile
DAPS	4,4′-Diamino diphenyl disulfide
DMA	Dynamic mechanical analysis
DTG	Differential thermogravimetry
EMA	Ethylene methacrylate
FTIR	Fourier-transform infrared
HDI	Hexamethylene diisocyanate
IPDI	Isophorone diisocyanate
IPN	Interpenetrating polymer network
MD	Molecular dynamics
MDI	Diphenylmethane diisocyanate
MOCA	3,3′-dichloro-4,4′-diaminodiphenylmethane
PEMA	Poly(ethylene methacrylate)
PMMA	Poly(methyl methacrylate)
PTMG	Poly(tetrahydrofuran ether) glycol
PU	Polyurethane
RDF	Radial distribution function
SEM	Scanning electron microscopy
TDI	Toluene diisocyanate
TGA	Thermogravimetric analysis
